# TIM-3+ Macrophages: Insights into Their Role in Cancer and Inflammation

**DOI:** 10.3390/ijms27020840

**Published:** 2026-01-14

**Authors:** Aleksandra Maksimova, Tamara Tyrinova, Elena Chernykh

**Affiliations:** 1 Laboratory of Cellular and Molecular Mechanisms of Immunopathology, Federal State Budgetary Scientific Institution Research Institute of Fundamental and Clinical Immunology, Novosibirsk 630099, Russia; parkinson.dses@gmail.com; 2 Laboratory of Cellular Immunotherapy, Federal State Budgetary Scientific Institution Research Institute of Fundamental and Clinical Immunology, Novosibirsk 630099, Russia; ct_lab@mail.ru

**Keywords:** TIM-3, macrophage, immune check point, oncology, pregnancy

## Abstract

T-cell immunoglobulin and mucin domain 3 (TIM-3), a well-known immune checkpoint molecule, is increasingly recognized for its regulatory functions beyond T cell exhaustion, particularly in macrophages. Recent advances have revealed the important role of this molecule in various pathological and physiological conditions. The demand for a comprehensive study of TIM-3 is increasing, particularly as a result of ongoing clinical trials targeting TIM-3 in oncology. This review is devoted to the role of TIM-3 in macrophage biology, focusing on associations between TIM-3 expression and macrophage polarization states and functional activity, as well as its involvement in the pathogenesis of different diseases and reproductive immunology. The review examines known effects and molecular mechanisms by which TIM-3 regulates macrophage functional phenotype and the contribution of TIM-3-expressing macrophages to cancer, pregnancy, inflammation, infectious and autoimmune diseases, and fibrosis. Findings highlight the controversial role of TIM-3 in the regulatory function of macrophages and suggest that TIM-3 functions differently depending on the context. The review also touches on gaps and unexplored parts of the topic. A summary of current data allows us to conclude that TIM-3 is an important modulator of macrophage functions and can be considered a potential therapeutic target in various pathological conditions.

## 1. Introduction

T-cell immunoglobulin and mucin domain 3 (TIM-3) are immune checkpoint receptors that are important for modulation of immune reactions. Checkpoint receptors are important players in the immune response that regulate T-cell activation. Their biological function is to downregulate signaling pathways, resulting in decreased production of pro-inflammatory cytokines and increased production of regulatory and anti-inflammatory cytokines by immune cells, as well as inhibition of proliferation and activation of effector T cells. All this contributes to the involvement of checkpoint receptors in immune tolerance, autoimmunity, and antitumor or antiviral immune evasion. The crucial role of these molecules in tumor immune evasion has led to the development of anti-programmed cell death protein 1 (PD-1) and anti-cytotoxic T-lymphocyte-associated protein 4 (CTLA-4) therapies for cancer. Currently, anti-TIM-3 drugs are being developed and are in clinical trials [1], since numerous preclinical studies show that TIM-3 expression on tumor cells leads to the development of resistance to anti-PD-1 or anti-CTLA-4 antibodies, and TIM-3 blocking induces an anti-tumor immune response [2,3,4,5]. Some authors think that targeting TIM-3 could be a good way to get around resistance to anti-PD-1 therapy.

Studies have also demonstrated a significant role of TIM-3 in autoimmune diseases. The TIM-3-ligand axis plays a key role in the pathogenesis of a number of autoimmune diseases and chronic inflammatory conditions, such as systemic lupus erythematosus, rheumatoid arthritis, inflammatory bowel disease, multiple sclerosis, vasculitis, and asthma [6]. Moreover, TIM-3 is an important molecule for infectious immunity and regulation of the inflammatory response [7,8,9,10].

With regard to T cells, the role of TIM-3 has been described in sufficient detail, and the scientific community has reached a certain consensus. Meanwhile, an increasing amount of evidence has emerged in recent years showing that TIM-3 is also important for the activity of myeloid cells. It was found that TIM-3 regulates the functions of monocytes, macrophages, and dendritic cells. Moreover, some studies provide information that TIM-3 expression on myeloid cells is higher than on T cells in some conditions [11,12,13].

TIM-3 is believed to have great significance in macrophage activity modulation. Since macrophages are multifunctional cells regulating various processes in the body, the issues of TIM-3 expression and signaling pathways triggered by the interaction of this molecule with ligands have become important. Macrophages are very flexible cells that are found in most tissues. They help control inflammation, repair, the strength of the immune response, and other normal and abnormal processes [14]. The role of tumor-associated macrophages is also well-known [15]. Therefore, clarifying the mechanisms of macrophage regulation is of particular interest since it increases the potential for using macrophages to regulate the above-mentioned processes in the body.

Thus, given the above, elucidating the role of TIM-3 in macrophage regulation is of particular interest, as it may open new therapeutic avenues for modulating immune responses in cancer and inflammatory diseases. Although TIM-3 is considered crucial for macrophage polarization and functional phenotype formation [16], current data on its role are controversial and fragmented. The precise mechanisms by which TIM-3 signaling regulates macrophage polarization and functions, and how this contributes to specific pathologies, remain uncertain and sometimes a subject of conflicting data. This review summarizes studies devoted to the importance of TIM-3 in the modulation of macrophage function and the role of TIM-3^+^ macrophages in various pathologies.

## 2. TIM-3 Structure and Ligands

**Figure 1:**
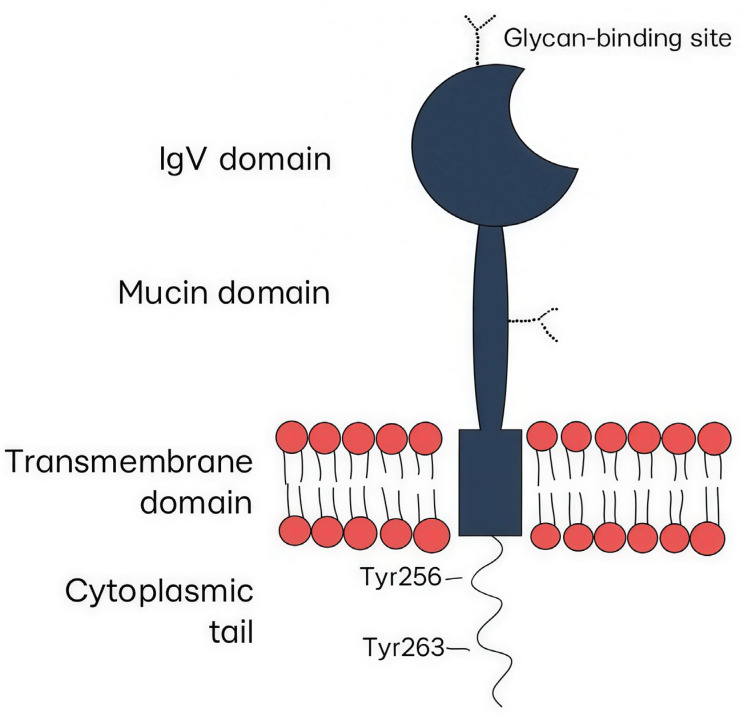
The TIM-3 structure includes a membrane-distal N-terminal immunoglobulin (IgV) domain, a mucin domain with N-linked glycosylation, a transmembrane domain, and a cytoplasmic tail.

TIM-3 belongs to the immunoglobulin superfamily and is widely expressed on the surfaces of adoptive and innate immunity cells. The TIM-3 structure includes a membrane-distal N-terminal immunoglobulin (IgV) domain and a mucin domain with N-linked glycosylation, which account for the extracellular part of the receptor, a transmembrane domain, and a cytoplasmic tail (Figure 1) [17]. The IgV domain has a specific structure because of extra disulfide bonds between noncanonical cysteines, which is a feature of the TIM superfamily. Engagement of the IgV domain with appropriate ligands is considered to provide the immunoregulatory role of TIM-3 [17].

TIM-3 exists in membrane-bound form and soluble form, which lacks the mucin and transmembrane domains. The precise mechanism of releasing soluble TIM-3 is still unknown; however, soluble TIM-3 is most likely the result of shedding by disintegrin and metalloprotease (ADAM)-10 and ADAM-17 [18,19].

Several ligands for TIM-3 are known at present, including galectin-9 (Gal-9) [20], phosphatidylserine (PtdSer) [6,21,22], high-mobility group protein B1 (HMGB1, amphoterin) [23,24] and carcinoembryonic antigen-related cell adhesive molecule 1 (CEACAM1) [25]. There are still many unresolved questions regarding the interaction between ligands and TIM-3. First, it is unclear whether all of the above-mentioned molecules are true TIM-3 ligands, since some studies have not linked TIM-3 function to a particular TIM-3 ligand [26]. Besides, Gal-9 and CEACAM-1 can regulate T cells and macrophages independently of TIM-3 [27,28,29,30,31,32]. Moreover, De Sousa Linhares et al. found that CEACAM1 does not bind to TIM-3 [32]. The second question is the exact binding sites of TIM-3 and the ligands, and whether there is any competition between TIM-3 ligands. Finally, some authors believe that TIM-3 can bind to several ligands at the same time. Thus, engagement with TIM-3 and Gal-9 does not exclude binding to other ligands at different binding sites [33]. Moreover, it is likely that the binding of Gal-9 to TIM-3 can lead to a change in the receptor configuration, resulting in the cleft framed by the FG and CC′ loops becoming more available to other ligands [33].

## 3. The Role of TIM-3 in Macrophages

### 3.1. TIM-3 Expression on Different Macrophage Subtypes

The TIM-3 plays a key role in regulating macrophage function, affecting macrophage polarization, cytokine profile, and effector functions. It acts as an immunological ‘switch’, and its effect depends on the microenvironmental context, the type of ligand, and the state of cells. This regulation is particularly important in light of the existence of different functional states in macrophages, the extreme points of which are pro- and anti-inflammatory phenotypes.

Macrophages are cells representing a spectrum of functional states, the opposite of which are M1 and M2, based on their expression of certain surface receptors and the secretion of specific molecules [34]. M1 cells are characterized by a pro-inflammatory phenotype; they express CD80, CD86, CD40, and inducible nitric oxide synthase (iNOS), produce pro-inflammatory cytokines (IL-1β, TNFα), and provide anti-infective and anti-tumor activity [34]. In contrast, M2 are characterized by anti-inflammatory activity, expressing arginase-1, CD206, CD163, and CD204, producing anti-inflammatory cytokines (IL-10, transforming growth factor TGF-β); they limit inflammatory response and promote tissue repair and wound healing [34]. Researchers are trying to compare TIM-3 expression and macrophage functional phenotypes in order to determine whether TIM-3 is linked to any specific phenotype and whether it might serve as a marker, and also identify the signaling pathways that are responsible for this.

Most studies assume a link between TIM-3 and the M2 polarization state of macrophages. Thus, an increase in TIM-3 expression was detected on M2 macrophages in mice [35,36]. Ocaña-Guzman et al. demonstrated that TIM-3 expression is higher on human monocyte-derived macrophages polarized with IL-4 (M2) compared to cells polarized with lipopolysaccharide (LPS)+IFNγ (M1) [37]. The association between TIM-3 levels and M2 polarization was also shown in the study of Sun J et al., in which mouse adipose tissue macrophages were biased toward the M2 phenotype with increased TIM-3 expression and, conversely, toward the M1 phenotype with decreased TIM-3 expression [38]. TIM-3 expression was increased on promonocytic THP-1 cells, which exhibit an M2-like phenotype when cultured with secreted factors from anaplastic thyroid cancer cells [39]. Zhang et al. found increased expression of TIM-3 on CD68^+^CD163^+^ macrophages (M2 phenotype) in rats [40]. Finally, IL-4/signal transducer and activator of transcription (STAT) 6 signals responsible for M2 polarization may promote TIM-3 activation [41]. Furthermore, Cheng et al. recently showed that the addition of Gal-9 led to a significant increase in the proportion of TIM-3^+^M1 and TIM-3^+^M2 macrophages and a decrease in M1 cell proportions and M1/M2 ratio [42].

On the other hand, Yu et al. indicated an association between TIM-3 and M1 polarization of macrophages during intracerebral hemorrhage. The authors demonstrated that intracerebral hemorrhage promoted TIM-3 expression and M1 polarization in the perihematomal region in mice. In contrast, TIM-3 knockout attenuated M1 polarization [43]. In addition, when studying decidual macrophages, the authors demonstrated that neither TIM-3^+^ nor TIM-3^−^ cells belonged exactly to either the M1 or M2 phenotype [44]. Thus, the link between TIM-3 and M1/M2 polarization probably depends on the localization of cells and the microenvironment, and it is not possible to unambiguously associate TIM-3 with a pro- or anti-inflammatory macrophage phenotype.

### 3.2. TIM-3 Signaling Pathways in Macrophages

**Figure 2:**
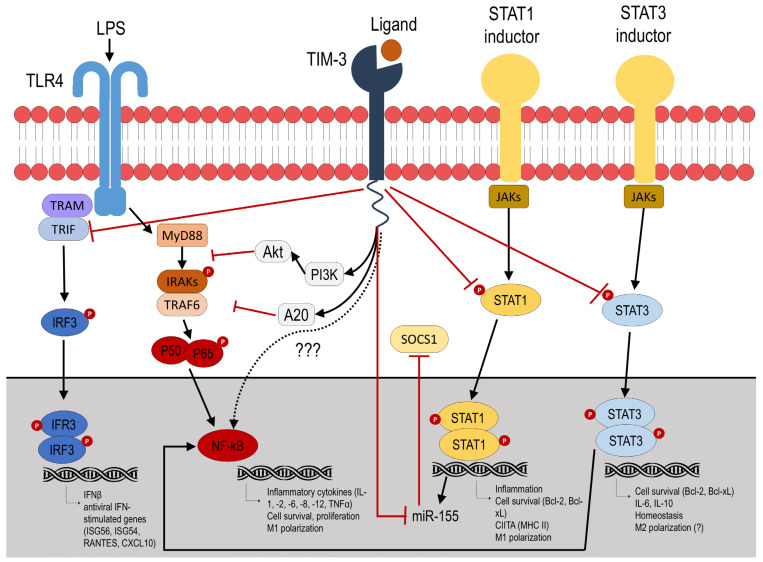
Described TIM-3 signaling mechanisms in macrophages. Question marks indicate that there is insufficient data on the given issue. Black arrows indicate the activating effect, red arrows indicate the inhibitory effect.

The exact mechanisms of TIM-3 effects are not yet fully understood. Due to non-canonical signaling, multiple ligands, and widespread expression, the TIM-3 pathway is very intricate and precisely regulated. It is still unknown exactly how TIM-3 regulates the pro- and anti-inflammatory activity of macrophages, although several studies have shown possible signaling cascades. TIM-3 acts through several intracellular pathways that can have both suppressive and activating effects on the inflammatory response (Figure 2).

One such mechanism is LPS/toll-like receptor (TLR) 4 signaling inhibition. TLR4 is known to transmit signals through two distinct pathways: the MyD88-dependent pathway, which mediates classical NF-κB activation, and the TRIF (TIR (Toll/interleukin-1 receptor) domain-containing adaptor protein inducing interferon beta)-dependent one, which is associated with interferon regulatory factor 3 (IRF3) [45]. The available evidence suggests that TIM-3 activation in macrophages is capable of inhibiting both of these signaling cascades.

Thus, it has been demonstrated that TIM-3 signaling inhibited LPS–TLR-4-mediated NF-κB activation, presumably by increasing PI3K–AKT phosphorylation and A20 activation [46]. Another recent study confirmed this fact and showed that TIM-3 suppressed inflammatory signaling pathway of p65, a protein involved in NF-κB heterodimer formation, nuclear translocation, and activation [47].

Additionally, TIM-3 signaling inhibited the phosphorylation of IRF3 [48], whereas TIM-3 knockout attenuated M1 polarization, presumably through stimulation of TRIF and IRF3 transcription factors [42]. Given that TRIF/IRF3 is associated with TLR-4, this may provide further support for the hypothesis that TIM-3 activation in macrophages suppresses TLR-4 pathway.

On the other hand, several studies produced opposite results. Under certain conditions, ligand-dependent activation of TIM-3 may, on the contrary, enhance TLR-4-mediated signaling and activation of the NF-κB/TNFα pathway, promoting a proinflammatory response. Thus, the activating effect of TIM-3 on TLR-4 signaling was found for intestinal macrophages [48]. The study demonstrated that TIM-3 blockade did not stimulate macrophage polarization in the M1 direction in TLR-4 knockout mice, unlike in wild-type mice. Moreover, the authors found the same effect on RAW264.7 mouse cell line macrophages. Another study showed that TIM-3-expressing renal macrophages aggravate the progression of diabetic nephropathy through activation of the NF-κB/TNFα pathway [49]. These facts partially contradict previous studies showing that TIM-3 negatively regulates TLR-4 and NF-κB signaling and require further research.

TIM-3 is also able to modulate macrophage polarization by influencing STAT proteins. Jiang et al. found that TIM-3 polarizes macrophages into M2 cells by directly binding to STAT1, which is associated with the M1 phenotype, and inhibiting the STAT1-miR-155-SOCS1 signaling axis [50]. The association of TIM-3 expression with STAT1 was also demonstrated in the study of Tang et al. The authors reported that TIM-3 can suppress major histocompatibility complex (MHC) II expression in macrophages through the STAT1/CIITA pathway [51]. In another study, TIM-3 suppression by T. gondii infection resulted in inhibition of PI3K-AKT phosphorylation, which mediates the transcription factors C/EBPβ and SOCS1. This led to increased proinflammatory markers and cytokines in decidual macrophages [52]. Axis PI3K-AKT is also associated with NF-κB pathway [53]; therefore, it may be an additional mechanism for NF-κB inhibition described above.

Ma et al. demonstrated that the cis-association of Gal-9 and TIM-3 inhibited STAT3 phosphorylation, which was accompanied by a decrease in IL-23p19 and an increase in IL-12p35 [54]. In turn, there is a close interaction between STAT3 and the NF-κB signaling [53]; therefore, these data may support the theory of NF-κB inhibition mediated by LPS/TLR-4 downregulation via TIM-3 activation.

At the same time, the relationship between TIM-3 and LPS (TLR4 inducer) is likely interdependent, and in addition to the effect of TIM-3 expression on TLR-dependent cytokine production, LPS and the LPS/TLR4 pathway can directly influence TIM-3 expression. Thus, it has been shown that TLR stimulation leads to a decrease in TIM-3 expression on CD14^+^ monocytes [55]. Interestingly, the effect of LPS on bone marrow-derived macrophages polarization via the TIM-3/Gal-9 pathway may be time-dependent. In the study by Zhang et al., short-term LPS stimulation activated the TIM-3/Gal-9 signaling pathway, which led to the inhibition of the proinflammatory M1 phenotype, whereas long-term LPS stimulation was accompanied by inhibition of the TIM-3/Gal-9 signaling pathway, ultimately leading to stimulation of M1 polarization [56]. The Gal-9/TIM-3 pathway partially activates cytokine production IL-6, IL-8, and IL-10, but not IL-1β, IL-12p70, or TNFα in response to LPS in gastric cancer tissue-derived tumor-associated macrophages, TAMs [57]. Taken together, this indicates ambiguous regulation of TIM-3 expression by LPS and may partially explain the differences described above.

In addition, TIM-3 expression may also depend on other TLRs. For example, Vidyarthi et al. showed that stimulation of macrophages by a TLR-3 ligand reduced the expression of TIM-3 on M2 macrophages and increased the release of proinflammatory cytokines [58].

In summary, TIM-3 signaling in macrophages is nonlinear and context-dependent. Its final effect—inhibitory or activating—is determined by a combination of factors: ligand type, the involved intracellular pathway, and the microenvironment (e.g., the presence of LPS, cytokine background). This plasticity allows TIM-3 to act as a fine regulator of the immune response, participating both in limiting excessive inflammation and, under certain conditions, in maintaining a proinflammatory environment. Resolving apparent discrepancies in the data requires consideration of the specific biological context in which TIM-3 function is studied.

### 3.3. Association Between TIM-3 and Macrophage Functions

TIM-3 has a complex effect on the condition and function of macrophages. First of all, by modulating the repertoire and balance of secreted cytokines, TIM-3 directs the functional outcome of macrophage activation.

The TLR-4/NF-κB pathway is known to be responsible for the secretion of proinflammatory cytokines, including IL-1, -2, -6, -8, -12, and TNFα [53]. Since TIM-3 likely acts as an inhibitor of the TLR-4/NF-κB axis, its regulatory effect on macrophage polarization is mediated, at least in part, through the direct suppression of this cytokine program. Indeed, blocking anti-TIM-3 antibodies and TIM-3 gene silencing resulted in increased production of IL-6, IL-12, TNFα, and IL-1β by macrophages and a decrease in M2 polarization, while overexpression of TIM-3 suppressed the production of these cytokines [46,47,59,60,61]. At the same time, Wang et al. showed that the interaction of Gal-9 with TIM-3 significantly stimulated the secretion of IL-6, IL-8, and IL-10, but not IL-1β, IL-12p70, or TNFα, in the presence of LPS [57], indicating an ambiguous influence of the Gal-9/TIM-3 pathway.

In turn, the LPS/TLR-4 pathway can also drive the production of IL-10, the powerful anti-inflammatory cytokine, as well [57,62,63]. Therefore, suppression of TLR-4 pathway by TIM-3 may explain increased IL-10 production after the TIM-3 suppression [46,49]. This mechanism may determine some of the pro-inflammatory signals of TIM-3, for example, that were found in the study by Anderson et al., where TIM-3 exerted a proinflammatory effect in the presence of a TIM-3 monoclonal agonist [64].

In addition to modulating the cytokine profile, TIM-3 is also likely involved in the phagocytic activity of macrophages. Thus, treatment with anti-TIM-3 antibodies inhibited the phagocytic activity of alveolar macrophages in vivo [65]. DeKruyff et al. also concluded that TIM-3 may partially mediate macrophage phagocytosis due to the ability to bind apoptotic cells [66]. In contrast, Liu et al. found that TIM-3 activation inhibits the phagocytic and cytotoxic effects of macrophages [47], and Hou et al. demonstrated that blocking anti-TIM-3 antibodies enhanced phagocytosis of red blood cells infected with the malarial pathogen by murine splenic macrophages [67].

Furthermore, TIM-3 is involved in the regulation of T cell functions by macrophages. For example, co-cultivation of TIM-3-overexpressing macrophages with intestinal lymphocytes reduced the proinflammatory response in a mouse model of colitis [48], and blocking TIM-3 on peritoneal macrophages resulted in a decrease in Tregs but an increase in Th1 and Th17 CD4^+^ T cells [51]. Li et al. showed that TIM-3^+^ macrophages caused a Th2 and Treg bias in CD4^+^ T cells in contrast to TIM-3^−^ macrophages and TIM-3 blockade [44].

Together, these findings suggest that TIM-3 exerts a complex effect on macrophage function by modulating cytokine secretion, phagocytic activity, and T-cell response. The contextual nature of these effects explains the dual and sometimes contradictory role of TIM-3, which must be considered when assessing its potential as a therapeutic target.

## 4. TIM-3 Expression on Macrophages in Cancer and Inflammation

### 4.1. Oncology

TIM-3 expression on macrophages and monocytes represents a significant mechanism of tumor evasion from immune surveillance, as supported by data obtained in various types of cancer. Macrophages in the tumor microenvironment (tumor-associated macrophages, TAMs) are able to express TIM-3. Thus, an increased level of TIM-3 expression on peripheral blood mononuclear cells and/or TAMs compared to healthy people was found in patients with hepatocellular carcinoma [68], colorectal cancer [11,69], non-small cell lung cancer [70], and gastric cancer [57]. Higher TIM-3 expression on macrophages was found in a patient who lost the sensitivity of spindle cell lung cancer to anti-PD-1 therapy after a good response [5]. TIM-3 was expressed on the surface of macrophages and microglia in patients with diffuse intrinsic pontine glioma (54%), whereas TIM-3 was not expressed in the brain of healthy individuals [71]. Vanmeerbeek et al. detected a niche of tumor-associated macrophages that co-expressed TIM-3 and V-domain immunoglobulin suppressor of T-cell activation (VISTA) [72].

TIM-3 expression on monocytes and macrophages may be one of the indicators of immune surveillance evasion of tumor. Hu et al. also demonstrated that high-level TIM-3 expression on the CD68^+^ cells (macrophage marker) indicates poor survival in glioma [73]. High TIM-3 expression in TAM was an independent predictor of poor prognosis in patients with non-small cell lung cancer and negatively correlated with overall survival [70]. In addition, increased TIM-3 expression on peripheral monocytes was associated with deeper tumor invasion, metastasis of tumor in the lymph nodes, and advanced clinical stage of cancer in patients with gastric cancer [57].

So, TIM-3-expressing macrophages are proposed as a potential target for anti-tumor therapy, and understanding their role and mechanisms of their activity in tumor pathology is crucial.

The negative effects of TIM-3-expressing macrophages are most often associated with the polarization of macrophages towards M2 and the occurrence of tumor-stimulating activity [39,50,68,69,74,75]. This process ensures a decrease in the production of proinflammatory cytokines (IL-12, TNFα, IL-6) and an increase in the production of IL-10 and TGF-β [50,74]. Additionally, as demonstrated for monocytes, TIM-3 expression on the cell surface can decrease the generation of IFNγ by activated CD8^+^ T cells [76].

Ni X. et al. showed that the interaction of Gal-9 and TIM-3 on macrophages leads to increased secretion of M2-associated proangiogenic factors such as vascular endothelial growth factor VEGF-A, while blockade of Gal-9/TIM-3 signaling inhibits M2 polarization and suppresses tumor growth in glioblastoma models [75].

**Figure 3:**
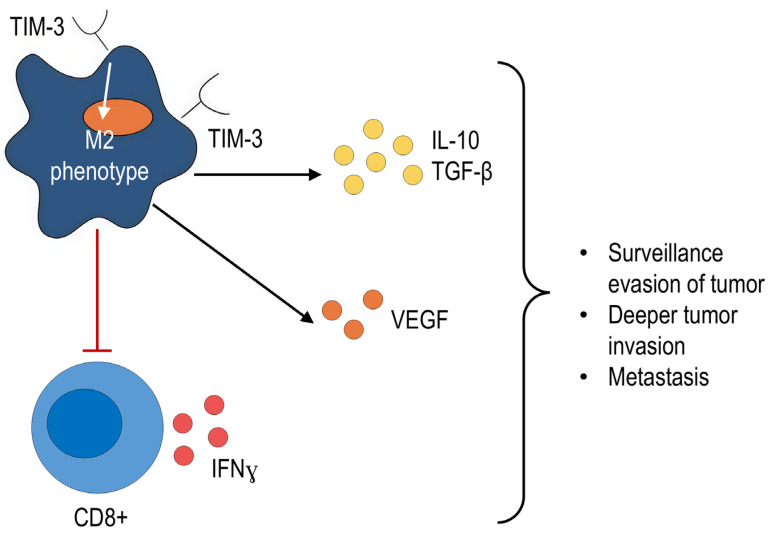
Described mechanisms of immunosuppression of TIM-3-expressing macrophages in oncology. Black arrows indicate the activating effect, red arrows indicate the inhibitory effect.

Thus, TIM-3 is associated with M2 polarization, production of anti-inflammatory cytokines and proangiogenic factors, and suppression of CD8^+^ T cell activity (Figure 3). These findings suggest that TIM-3 blockade could be a promising strategy for overcoming resistance to the available checkpoint inhibitor treatments and explain the interest of scientists in developing new checkpoint inhibitors.

At the same time, as demonstrated in Section 3.2 and Section 3.3, TIM-3 signaling in macrophages has a complex, contradictory biology, modulating both pro- and anti-inflammatory pathways. This duality suggests that TIM-3 blockade may have unintended consequences, highlighting the necessity for comprehensive preclinical and clinical studies to accurately predict its effects and understand the full implications of this therapy.

### 4.2. Non-Infectious Inflammation

TIM-3 plays an important role in the regulation of inflammation [76,77], but data on TIM-3 in myeloid cells are conflicting.

The anti-inflammatory effect of TIM-3 was described in a mouse model of acute inflammation. Wang et al. demonstrated that TIM-3-knockout mice showed increased susceptibility to colitis as well as more severe colitis and increased expression of proinflammatory mediators compared with controls [59]. Jiang et al. also showed that TIM-3 inhibits the polarization of pathogenic proinflammatory M1 macrophages in colitis, whereas TIM-3 down-regulation or blockade leads to an increase in the M1 response [48]. Moreover, adoptive transfer of TIM-3-silenced macrophages aggravates DSS (dextran sulfate sodium)-colitis and increases inflammation, while co-culture of TIM-3-overexpressing macrophages with intestinal lymphocytes reduces the pro-inflammatory response [48].

TIM-3 may also be involved in the pathogenesis of chronic diseases. For example, TIM-3 exerted a protective effect in steatohepatitis in mice by limiting the secretion of reactive oxygen species and inflammatory cytokines [46].

On the other hand, TIM-3 expression in microglia/macrophages was found to enhance inflammation after intracerebral hemorrhage and was positively correlated with pro-inflammatory cytokine (TNFα, IL-1β) concentrations and brain water content [43,77].

In another study, TIM-3 overexpression on myeloid cells led to the development of excessive lung inflammation in a mouse model, which was associated with galectin-3 production. TIM-3 blockade or galectin-3 inhibition was associated with a reduced inflammatory response in TIM-3 overexpressing mice. However, TIM-3 knockout resulted in lower body weight and shorter lifespan than WT mice, partly suggesting a positive role for TIM-3 in inflammation [78].

**Figure 4:**
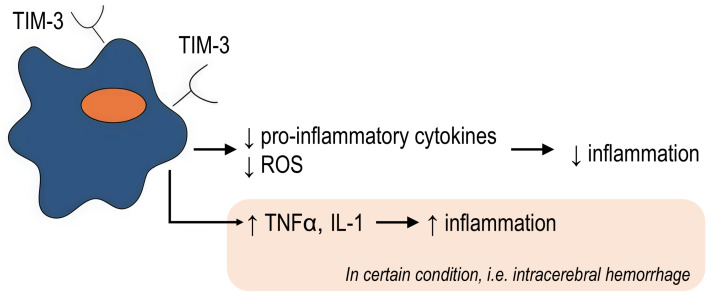
The dual and context-dependent role of TIM-3^+^ macrophages in inflammation. ↑ symbol indicates up-regulation, ↓ symbol indicates down-regulation.

Taken together, the obtained data indicate a context-dependent function of TIM-3 during inflammation (Figure 4). On the one hand, TIM-3^+^ macrophages are associated with the suppression of acute and chronic inflammation; on the other hand, some studies have demonstrated that TIM-3^+^ macrophages can support the inflammatory response. These differences may be due to the macrophage origin, niche, and the cause of the inflammatory response.

### 4.3. Fibrosis as a Result of Unresolved Inflammation

**Figure 5:**
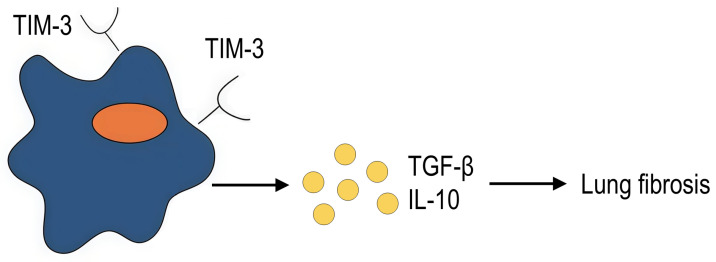
TIM-3-expressing macrophages aggravate pulmonary fibrosis (summary of the study by Wang et al. [79]).

Inflammation is a complex process that, when dysregulated, can drive fibrosis. Given the crucial role of TIM-3-expressing macrophages in modulating inflammation, they are likely implicated in the fibrotic process as well. However, direct evidence linking TIM-3^+^ macrophages to fibrogenesis remains limited. To date, there is only one study examining the role of TIM-3^+^ macrophages in fibrosis. Wang et al. showed that TIM-3 aggravates pulmonary fibrosis. TIM-3 transgenic mice developed more severe pulmonary fibrosis compared to wild-type mice, but after macrophage depletion, the fibrosis score was significantly reduced in both wild-type and TIM-3 transgenic mice. Adoptive transfer of macrophages derived from TIM-3 transgenic mice resulted in more severe fibrosis compared to transfer of macrophages derived from wild-type mice. In light of these observations, the authors conclude that TIM-3-expressing macrophages exacerbate pulmonary fibrosis, probably due to increased production of profibrogenic markers of pulmonary fibrosis, IL-10 and TGF-β [79] (Figure 5). In turn, these factors can exert both profibrogenic and anti-inflammatory effects, which confirms the context-dependent involvement of TIM^+^ macrophages in the inflammatory process.

### 4.4. Autoimmune Inflammation

Previous studies have shown that TIM-3 dysfunction influences various immune cells and contributes to the pathogenesis of autoimmune disorders [6]. Studies conducted on T cells have shown that reduced TIM-3 expression in peripheral blood mononuclear cells may serve as an unfavorable prognostic factor in multiple sclerosis [80]. Anti-TIM-3 antibodies promote IFNγ and MCP-1 secretion by synovial mononuclear cells in patients with rheumatoid arthritis [81].

**Figure 6:**
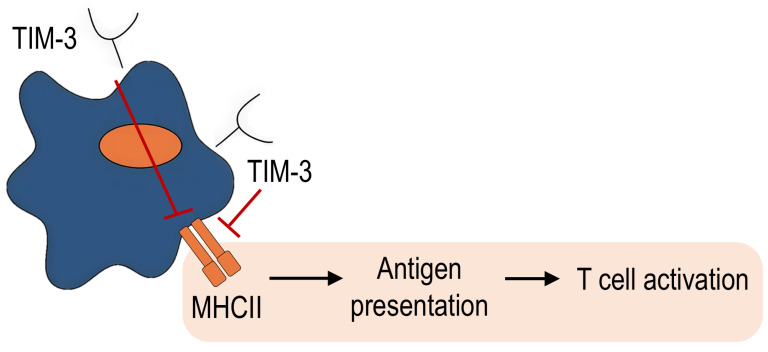
The possible mechanism of involvement of TIM-3^+^ macrophages in autoimmune inflammation pathogenesis.

However, the role of TIM-3 expression on macrophages in autoimmune diseases is not clearly known, which is probably associated with the more important role of T cells in the pathogenesis of these diseases. Only one study investigating the role of TIM-3 expression on macrophages in autoimmune encephalomyelitis in mice showed that TIM-3 overexpression led to a decrease in inflammation, demyelination areas, and disease score in mice. At the same time, TIM-3 blocking, on the contrary, led to a significant increase in the severity of encephalomyelitis. These effects were associated with the ability of TIM-3 to suppress MHC-II expression in macrophages, which led to a decrease in antigen presentation and subsequent T cell activation (Figure 6) [51]. These findings suggest that TIM-3^+^ macrophages apparently play a positive role in autoimmune inflammation. However, further investigation is required to draw definitive conclusions, including studies of other autoimmune diseases such as systemic scleroderma, rheumatoid arthritis, ankylosing spondylitis, and systemic lupus erythematosus.

### 4.5. Infections

TIM-3 is involved in the regulation of anti-infective immune response [82]. Thus, after TIM-3 and PD-1 blocking, macrophages had a higher ability to limit the growth of *M. tuberculosis* in HIV+ (human immunodeficiency virus) patients [83]. In addition, some infectious agents induce infectious tolerance by increasing TIM-3 expression on macrophages, such as *Schistosomes* or *Plasmodium*. Moreover, blockade of TIM-3 during *Schistosome* infection led to polarization of macrophages in the antibacterial M1 direction, accompanied by increased expression of iNOS and IL-12 as well as a decrease in Arg1 and IL-10 [84]. Similarly, blockade of the TIM-3 signaling with anti-TIM-3 antibodies during *Plasmodium* infection enhanced phagocytosis and production of antiparasitic mediators by macrophages [67]. TIM-3 signaling is shown to suppress the phagocytic and cytotoxic functions of macrophages against *C. albicans* [47]. In addition, Tim-3 negatively regulates the secretion of IL-12 by monocytes in *Hepacivirus C* infection [85].

On the other hand, the anti-inflammatory action of TIM-3 appears to help restrict tissue damage in acute and chronic inflammation. Overexpression of TIM-3 in murine RAW264.7 macrophages reduced *H. pylori*-associated inflammation by blocking TLR4 and secretion of proinflammatory cytokines [86]. On monocytes, the findings are consistent with the broader hypothesis that TIM-3 helps suppress chronic infection, such as herpesvirus infection [87].

Yang et al. demonstrated that TIM-3 plays an important role in limiting sepsis-induced inflammation in both humans and mice. Downregulation and/or blockade of TIM-3 correlated with the severity of sepsis. Blockade and/or reduction in TIM-3 expression resulted in increased macrophage activation, which contributes to the systemic inflammatory response in sepsis. TIM-3 overexpression in macrophages significantly suppressed the production of proinflammatory cytokines [46].

On the other hand, TIM-3 expression may, under certain conditions, enhance the proinflammatory activity of myeloid cells. Thus, activation of TIM-3 in monocytes from patients with chronic hepatitis B increased the production of TNFα, IL-1β, and IL-6, while blocking TIM-3 reduced the ability of monocytes to induce a Th17 response [88].

**Figure 7:**
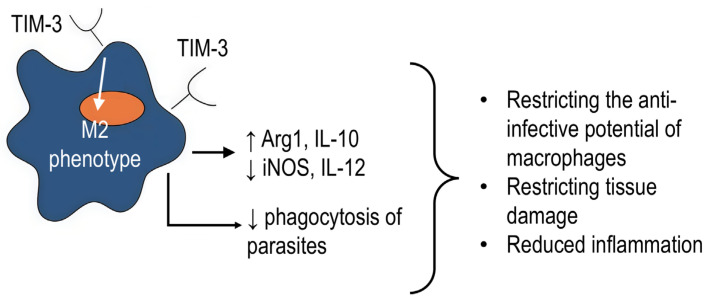
Mechanisms and effects of TIM-3 in anti-infective immunity of macrophages. ↑ symbol indicates up-regulation, ↓ symbol indicates down-regulation.

Therefore, the cumulative evidence strongly suggests that TIM-3 on monocytes/macrophages predominantly plays a negative role in infection by restricting the anti-infective potential of macrophages (Figure 7). This negative regulation manifests likely through the promotion of M2-like phenotype, the attenuation of pro-inflammatory signaling pathways (e.g., TLR4), and further direct suppression of macrophage effector functions, such as phagocytosis and the production of antimicrobial mediators. While it might be beneficial in preventing persistent inflammation during certain chronic infections, it facilitates survival and persistence of various infectious agents within the host. Besides, TIM-3 in some infections may have a stimulatory role in inflammatory cytokine production and Th17 cell response.

### 4.6. Diseases Associated with Inflammation in Pregnancy

Pregnancy is a unique condition in which the balance of pro- and anti-inflammatory agents is crucial. Disbalance leads to pathological processes such as preeclampsia and recurrent miscarriage [89]. It is noteworthy that TIM-3 is a critical immune checkpoint and multifunctional immunoregulatory molecule during pregnancy, inducing maternal-fetal tolerance and harmonizing the interactions between different cells in the process of maternal-fetal tolerance [90].

TIM-3 is constitutively expressed on decidual macrophages [52], and its expression is also increased on monocytes in the peripheral blood during pregnancy [41,44]. It is believed to be an important checkpoint that induces fetomaternal tolerance during pregnancy. TIM-3 expression is crucial for preventing pregnancy loss. A decrease in TIM-3 on CD14^+^ cells in the peripheral blood and decidual cells is associated with an imbalance in the anti- and pro-inflammatory cytokine profile, dysfunction of fetomaternal tolerance, and miscarriage [44,91].

In mouse models, adoptive transfer of TIM-3^+^ macrophages, but not TIM-3^−^ macrophages, reduced macrophage depletion-induced abortion in mouse embryos [44,89], which may be associated with the production of angiogenic growth factors by TIM-3^+^ cells [92].

Preeclampsia-like disorders in rats are attenuated via the TIM-3/Gal-9 pathway, which reduces inflammatory cytokine levels [93].

On the other hand, other studies have suggested that abnormal TIM-3 expression levels may be associated with pregnancy loss [41]. One recent study found that significantly elevated TIM-3 levels on maternal macrophages in the decidua may be associated with the development of preeclampsia. However, the authors found this association for the female fetus but did not confirm it in the overall sample. In addition, the differences were on the very fringes of significance (*p* = 0.049) [94].

**Figure 8:**
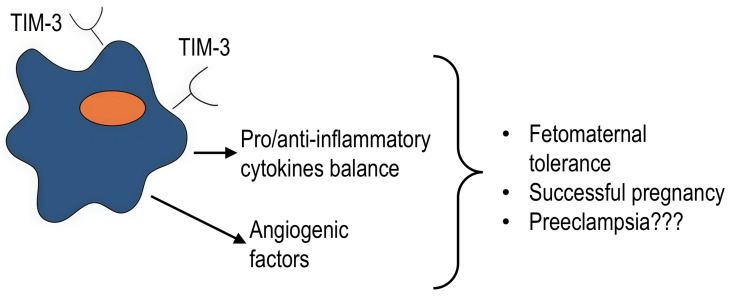
TIM-3 regulates the balance of pro- and anti-inflammatory cytokines during pregnancy. Its role in preeclampsia remains poorly understood. Question marks indicate that there is insufficient data on the given issue.

Therefore, TIM-3 expression on macrophages is an important regulator of successful pregnancy, fine-tuning the critical balance between pro- and anti-inflammatory states at the fetomaternal interface. These protective effects are likely mediated by angiogenic factor secretion and suppression of inflammatory cytokines (Figure 8); however, distinct mechanisms remain to be seen. Future research must focus on defining the optimal “therapeutic window” of TIM-3 activity and the distinct signaling pathways it engages in different pathologies of pregnancy.

## 5. Conclusions

There is still much to learn about such a unique molecule as TIM-3. However, its expression on myeloid cells and, in particular, macrophages, plays a critical role in the regulation of the functional activity of these cells.

Despite a large amount of data demonstrating the anti-inflammatory effect of TIM-3 signaling and its association with the M2 phenotype of macrophages, some studies have found a link between TIM-3 expression and the proinflammatory properties of macrophages. Therefore, TIM-3 cannot be classified definitely as an inhibitory molecule since it rather plays a regulatory role.

The issue of ligand interactions with TIM-3 and TIM-3 signaling cascades remains unresolved. The exact pathways of TIM-3 signaling in macrophages require determination, but it is most likely that it is associated with TLRs and, in particular, TLR-4. Some of the probable mechanisms include the NF-κB, IRF3, STAT1, and STAT3 pathways. Further research in this area should clarify the discrepancies found and establish the exact pathways of activation of the TIM-3.

TIM-3 expression on macrophages is important for cancer and infectious diseases, acute and chronic inflammatory processes, as well as some physiological conditions, such as pregnancy. Given the ongoing clinical trials of anti-TIM-3 monoclonal antibodies for different diseases, the study of TIM-3 expression in macrophages, signaling pathways, and effects on macrophage functions is still relevant and necessary.

Current evidence indicates that TIM-3^+^ macrophages apparently play a negative role in oncological diseases, whereas their function in inflammation is context-dependent, and TIM-3 can either limit or promote the inflammatory response. These insights indicate that TIM-3^+^ macrophages represent a promising target for the development of further therapies aimed at illnesses such as cancer and both acute and chronic inflammation. Furthermore, TIM-3 may be a potential target in infectious diseases, as it appears to suppress anti-infective immunity. During pregnancy, the effect of TIM-3-expressing macrophages is likely positive; however, studies demonstrating the negative effect of TIM-3^+^ macrophages require further research to elucidate the role of TIM-3 during pregnancy.

The involvement of TIM-3^+^ macrophages in autoimmune inflammation and in defective inflammation resolution, particularly in fibrosis, is poorly understood and necessitates additional investigation. The staging of inflammation and different pathogenesis may be key factors in the divergence of TIM-3^+^ macrophage functions.

A deeper understanding of the physiopathological role of the TIM-3 pathway in innate immunity will shed new light on the pathogenesis of clinical diseases such as autoimmune diseases, chronic viral infections, and cancer, and suggest new approaches to intervention. The TIM-3 ligand axis and downstream signaling pathway molecules may become pivotal targets for future treatments.

## Data Availability

No new data were created or analyzed in this study. Data sharing is not applicable to this article.

## Abbreviations

The following abbreviations are used in this manuscript:

TIM-3	T-cell immunoglobulin and mucin domain 3
PD-1	Programmed cell death protein 1
CTLA-4	Cytotoxic T-lymphocyte-associated protein 4
Ig	Immunoglobulin
ADAM A	disintegrin and metalloprotease
Gal-9	galectin-9
PtdSer	Phosphatidylserine
HMGB1	High-mobility group protein B1
CEACAM1	Carcinoembryonic Antigen-Related Cell Adhesive Molecule 1
STAT	Signal transducer and activator of transcription
LPS	Lipopolysaccharide
CD	Cluster of differentiation
iNOS	Inducible nitric oxide synthase
IL	Interleukin
TNFα	Tumor necrosis factor α
TGF-β	Transforming growth factor β
IFNγ	Interferon gamma
THP-1	Human pro-monocytic cell line (acute monocytic leukemia)
TLR	Toll-like receptor
NF-κB	Nuclear factor kappa-light-chain-enhancer of activated B cells
IRF	Interferon regulatory factor
TRIF	TIR (Toll/interleukin-1 receptor) domain-containing adaptor protein inducing interferon beta
SOCS	Suppressor of cytokine signaling proteins
VEGF	Vascular endothelial growth factor
RAW264.7	Mouse macrophage cell line (tumor tissue induced by Abelson murine leukemia virus)
TAM	Tumor-associated macrophages
DSS	Dextran Sulfate Sodium
MHC	Major histocompatibility complex
HIV	human immunodeficiency virus
